# Editorial: Multimodal magnetic resonance imaging methods to explore the visual pathway and brain network changes in blindness disease

**DOI:** 10.3389/fnhum.2023.1143465

**Published:** 2023-02-03

**Authors:** Zhi Wen, Yan Tong, Xin Huang

**Affiliations:** ^1^Department of Radiology, Renmin Hospital of Wuhan University, Wuhan, China; ^2^Department of Ophthalmology and Visual Sciences, Faculty of Medicine, The Chinese University of Hong Kong, Shatin, Hong Kong SAR, China; ^3^Department of Ophthalmology, Jiangxi Provincial People's Hospital, The First Affiliated Hospital of Nanchang Medical College, Nanchang, Jiangxi, China

**Keywords:** blindness disease, visual cortex, brain network, fMRI, DTI, ASL

Diseases leading to blindness, such as glaucoma, diabetic retinopathy, optic neuritis and hereditary optic neuropathy, are a global problem. These diseases reduce visual acuity or are related to visual field defects, which results in severe morbidity in affected individuals.

Clinical imaging research related to blindness has shifted from the focus on the orbit to the changes in the structure and function of the brain. According to the symptoms and sensory dysfunctions of many eye diseases, the structure and function of the brain has been affected. FMRI is a non-invasive technique that can reveal these corresponding changes in the brain. It also provides a large amount of evidence of the structural and functional changes of the visual pathway after vision loss, and shows how the visual pathway is affected by eye diseases. More importantly, the development of algorithms and analysis enables us to discover new and valuable biomarkers from the current data. These advanced methods, combined with ophthalmological examination, enable researchers to infer the underlying mechanism of clinical manifestations from different aspects. It might provide new insights into the neuronal characteristics of visual impairment, identify the disease characteristics objectively and accurately, and provide promising therapies on this basis.

In this Research Topic, we collected 16 articles on a broad spectrum of blindness diseases. The authors introduced new imaging indicators, classified them by using machine learning, reviewed the literature and made contributions to this Research Topic. In this editorial, we give an overview of these different articles and group them according to the study design.

## Regional indictors

Hu et al. and Liang et al. explored the fractional amplitude of low-frequency fluctuation (fALFF) changes of patients with comitant exotropia and dry eye disease, respectively, compared to healthy controls (HC). ROC curve showed high diagnostic value of fALFF for distinguishing disease from HC.

Xiao et al. and Liu et al. examined the regional homogeneity (ReHo) changes in patients with retinal vein occlusion and with severe obesity and meibomian gland dysfunction, respectively. Fu et al. applied ReHo and functional connectivity (FC) method to assess the altered local and remote connectivity in primary angle-closure glaucoma (PACG). The accuracy of ReHo to distinguish PACG from HC is good by using the support vector machine (SVM) method.

Chen, Zhong, et al. and Wen et al. used the degree centrality (DC) and SVM method to determine the potential neuropathological mechanism of primary angle-closure glaucoma (PACG) and with early blindness caused by nystagmus, respectively. The accuracy of DC to distinguish PACG from HC is certain.

## Dynamic regional indictors

Ji, Cheng, et al. and Chen, Ye, et al. used ReHo and the amplitude of low-frequency fluctuation (ALFF) combined with sliding window method to evaluate the changes of dynamic neural activity in patients with high myopia and comitant exotropia, respectively, which was helpful to the diagnosis of the diseases. However, by using dALFF map as the classification feature and the SVM method, the discrimination of CE from HC was not good.

## Functional connectivity and large-scale network indictors

Homotopic connectivity as a key feature of the brain, is also a research hotspot. Qi et al., Cheng et al., and Chen, Hu, et al. applied voxel-mirrored homotopic connectivity (VMHC) method to evaluate the changes of FC between hemispheres in patients with thyroid-associated ophthalmopathy, high myopia, and comitant exotropia relative to HC, respectively. Moreover, Qi et al. and Chen, Hu, et al. took the VMHC as the classification feature, SVM method had achieved good performance in distinguishing patients from HC.

Ji, Shi, et al. used independent component analysis (ICA) to detect the changes of FC and functional network connectivity (FNC) within and between resting-state networks (RSNs) in patients with high myopia. It was found that the default mode network and cerebellar dysfunction were related to visual, cognitive and motor balance deficits.

## Literature review

Two literature reviews by Sujanthan et al. reviewed the efforts made by visually-driven fMRI (Sujanthan et al.) and rs-fMRI (Sujanthan et al.) on optic neuropathy, respectively, covered the changes of region activity, FC, and FNC. These papers emphasized the importance of multidimensional representation of optic neuropathy.

Last but not least, Sun et al. reported that clinical, radiologic characteristics of Leber's hereditary optic neuropathy (LHON) associated with multiple-related diseases, especially different subtypes of optic neuritis (ON), which were exhibited with idiopathic orbital inflammatory syndrome (IOIS) and compression optic neuropathy for the first time in this cohort. This condition may be a distinct entity with an unusual clinical and therapeutic profile.

In short, in the current Research Topics, most articles are devoted to describing the characteristics of blindness diseases in a single modality. We imagine that it is possible to integrate the information among multi-dimensional modalities and provide a comprehensive description of a specific disease in future. In addition, with the large-scale data, machine learning and artificial intelligence will help to understand the mechanism, to diagnose accurately, and to optimize treatment of blindness.

## Author contributions

ZW: writing original draft. YT and XH: writing review and editing. All authors contributed to the article and approved the submitted version.

